# A systems biology approach to suppress TNF-induced proinflammatory gene expressions

**DOI:** 10.1186/1478-811X-11-84

**Published:** 2013-11-07

**Authors:** Kentaro Hayashi, Vincent Piras, Sho Tabata, Masaru Tomita, Kumar Selvarajoo

**Affiliations:** 1 Institute for Advanced Biosciences, Keio University, 14-1 Baba-cho, Tsuruoka, Japan; 2 Systems Biology Program, Graduate School of Media and Governance, Keio University, Fujisawa, Endo, 5322, Japan

**Keywords:** TNF, Cell signaling, Computational model, Inflammation, RIP1

## Abstract

**Background:**

Tumor necrosis factor (TNF) is a widely studied cytokine (ligand) that induces proinflammatory signaling and regulates myriad cellular processes. In major illnesses, such as rheumatoid arthritis and certain cancers, the expression of TNF is elevated. Despite much progress in the field, the targeted regulation of TNF response for therapeutic benefits remains suboptimal. Here, to effectively regulate the proinflammatory response induced by TNF, a systems biology approach was adopted.

**Results:**

We developed a computational model to investigate the temporal activations of MAP kinase (p38), nuclear factor (NF)-κB, and the kinetics of 3 groups of genes, defined by early, intermediate and late phases, in murine embryonic fibroblast (MEF) and 3T3 cells. To identify a crucial target that suppresses, and not abolishes, proinflammatory genes, the model was tested in several *in silico* knock out (KO) conditions. Among the candidate molecules tested, *in silico* RIP1 KO effectively regulated all groups of proinflammatory genes (early, middle and late). To validate this result, we experimentally inhibited TNF signaling in MEF and 3T3 cells with RIP1 inhibitor, Necrostatin-1 (Nec-1), and investigated 10 genes (*Il6, Nfkbia, Jun, Tnfaip3, Ccl7, Vcam1, Cxcl10*, *Mmp3, Mmp13, Enpp2*) belonging to the 3 major groups of upregulated genes. As predicted by the model, all measured genes were significantly impaired.

**Conclusions:**

Our results demonstrate that Nec-1 modulates TNF-induced proinflammatory response, and may potentially be used as a therapeutic target for inflammatory diseases such as rheumatoid arthritis and osteoarthritis.

## Introduction

The tumor necrosis factor (TNF), first termed in 1962 [1], was initially known for its ability to induce programmed cell death or apoptosis. As a result, throughout the years, the TNF has been intensely investigated for its anticancer property [2]. Today, this cytokine is central to the regulation of myriad important cellular processes such as proliferation, differentiation, growth, and the immune response.

TNF binds to two types of outer membrane bound receptors on target cells, TNFR1 and TNFR2, and triggers the cell survival and proinflammatory NF-κB and MAP kinases activations [3]. In addition, the TNFR1 induces intracellular cell death pathways via caspases after internalization through endocytosis. It is, therefore, conceivable that the dysregulation of the TNF signaling process will misbalance proinflammatory and/or apoptotic responses. Notably, the chronic aberration in the baseline levels of TNF in human circulatory system has been attributed to the pathogenesis of numerous diseases, including rheumatoid arthritis, osteoporosis, sepsis and cancer [4,5].

The vast majority of TNF related biological processes are initiated by the death-domain (DD) containing TNFR1, which is also called TNFRSF1A. Unlike TNFR2, TNFR1 is present in almost all cell types in humans. Upon TNF binding, TNFR1 trimerizes, and its intracellular DD recruits TRADD, which then creates a platform for RIP1 and TRAF2 to collectively form the receptor-signaling complex I. Cellular inhibitor of apoptosis proteins (cIAP)-1 and -2 bind to complex I and, consequently, together with K63-linked ubiquitin chains, modify RIP1 and TRAF2 [6]. This creates docking sites for an E3 ligase or linear ubiquitin chain assembly complex (LUBAC) consisting of heme-oxidized IRP2 ubiquitin ligase-1 (HOIL-1), HOIL-1-interacting protein (HOIP), and SHANK-associated RH domain interacting protein (SHARPIN). Subsequently, the activation of TAK1 and the ubiquitination of NEMO (or IKKγ), a subunit of IKK complex, lead to cell survival or proinflammatory response through NF-κB and MAP kinases activations. Other TRAF superfamily members (TRAF5 and 6) are also known to play a role in the NF-κB and MAP kinases activations [7,8].

On the other hand, for the apoptotic pathways, clathrin, AP-2 and Dyn first mediate receptor internalization. Receptor-signaling complex I becomes modified, and dissociates from TNFR1, allowing FADD and caspase-8 to form complex II. Within complex II, caspase-8 becomes activated to induce extrinsic apoptosis through caspase-3 activation. Alternatively, caspase-8 activates caspase-7, and eventually, the cleavage of Bid to tBid in the mitochondria activates caspase-9 via cathepsin D. This induces the intrinsic apoptosis through caspase-3 activation.

Due to its ability to signal numerous cellular processes via the survival and death pathways, the TNFR1 signaling research has received immense attention over the years, especially on understanding the downstream signaling cascades to regulate and control proinflammatory diseases and cancer. Despite numerous studies, the control of proinflammatory diseases through therapeutic treatments, where TNF is over-expressed, remains suboptimal. For example, biologic response modifiers or biologics, such as Etanercept and Infliximab, are TNF decoy receptors or antibodies that suppress TNFR1 signaling through competition for TNF. Although these drugs have shown successful downregulation of inflammation in many cases, they can immuno-compromise patients to secondary infections such as tuberculosis [9], or have been ineffective in a substantial number of administered patients [10].

To find alternatives, there have been major efforts on selectively suppressing the intracellular signaling of TNFR1. For example, genetic knockouts (KOs) of TRAFs and TRADD acting on the proinflammatory pathways have been investigated [7,8,11]. However, the experimental outcomes, so far, have not been optimistic. In TRAF2 KO, there is compensatory activation of NF-κB through TRAF5 [7] or TRAF6 [8], and vice-versa. On the other hand, TRADD KO almost completely abolishes NF-κB activation [11], which is not desirable for the general survivability of cells. Thus, a systemic approach where the propagation of signal transduction to all known branching pathways during target intervention should be monitored. This will allow the elucidation of effective target candidate(s) that overcomes and balances the deficiencies of current investigations.

In this paper, we adopted a systems biology approach to study TNFR1 signaling dynamics. Firstly, we developed a computational model of TNF-induced proinflammatory response leading to NF-κB, MAP kinase activations, and three groups of gene expressions (classified according to their temporal profiles [12]). The model is based on the perturbation-response approach [13-16], which has been successfully used to elucidate novel signaling features and behaviors in Toll-like receptor-4 [17,18], -3 [19], and TNF-related apoptosis-inducing ligand (TRAIL) signaling [20]. Secondly, the TNFR1 model parameters were selected to fit the temporal activation profiles of NF-κB and MAP kinase p38 for fibroblast cell type in several available conditions (wildtype [7], TRAF2 KO [7], TRAF5 KO [7], TRAF2/TRAF5 double KO (DKO) [7], TRAF6 KO [8], TRADD KO [11] and RIP1 KO [21]). Using the resultant TNFR1 model with robust parameters, we performed simulations of multiple *in silico* KOs to determine an optimal target that suppresses, but not abolishes, proinflammatory genes. Finally, to validate the modeling results, we performed experiments measuring various key proinflammatory gene expressions in MEF and 3T3 cells for TNF stimulation. Overall, our study presents evidence that systems biology research can be useful to elucidate important target(s) to suppress proinflammatory diseases such as rheumatoid arthritis and osteoarthritis.

## Results

### TNFR1 signaling topology and model

**Figure 1:**
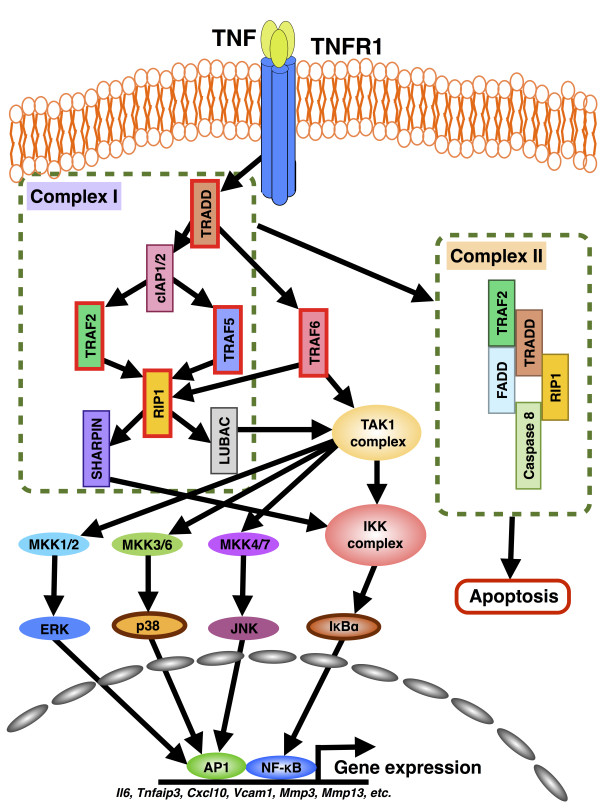
**Schematic of TNFR1 signaling of cell survival/proinflammatory and apoptosis pathways.** Upon TNF receptor activation, complexes I (survival pathway) and II (apoptosis) are formed. Complex I subsequently activates transcription factors, such as activator protein (AP)-1 and NF-κB through MAP kinases and IKK complex, respectively, which subsequently bind to promoter regions of genes to induce numerous proinflammatory genes.

To develop a computational model of proinflammatory TNFR1 signaling dynamics, we first require the known signal transduction pathways. We curated the KEGG database, and performed literature survey of the latest TNF research. After carefully considering several sources, we were able to propose a signaling topology mainly by combining the knowledge from KEGG, Falschlehner et al. (2012) and Wertz et al. (2010) [6,22] (Figure 1).

Next, to simulate TNF-induced dynamics of NF-κB and MAPK activations using the topology, we developed a dynamic model based on perturbation-response approach (Materials and Methods), using COPASI simulation platform [23]. Unlike common biochemical reaction models [24,25], the perturbation-response approach does not require detailed knowledge of all signaling species and their reaction kinetics. This is because it analyses the response waves of signal transduction instead of individual reaction kinetics [13-15,17-20]. The response waves can be approximated using linear response rules (*Response Rules*, Additional file 1: Figure S1) combined with the law of mass conservation, and this approach has been previously used to successfully model the TLRs and TRAIL signaling pathways [17-20].

Briefly, each reaction in the model is represented by a first-order response equation with activation or deactivation term. The activation term generally refers to protein binding, transformation, complex formation, phosphorylation and transcription. The deactivation term refers to protein unbinding, dephosphorylation and negative regulation such as mRNA decay through microRNA regulation.

### Simulating TNF-induced Ν F-κΒ and MAP kinase dynamics

**Figure 2:**
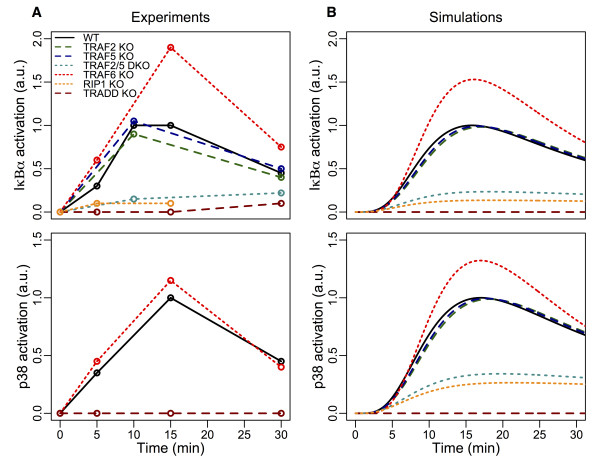
**Experimental and simulated profiles of IκBα and p38 activations in wildtype and mutant conditions. (A)** Experimental profiles, MEFs were generally treated with 10 ng/mL of TNF, and **(B)** simulated profiles of IκBα (top panels) and p38 (bottom panels) activations up to 30 min in wildtype (WT), TRAF2 KO, TRAF5 KO, TRAF2/TRAF5 double KO (TRAF2/5 DKO), TRAF6 KO, TRADD KO and up to 15 min in RIP1 KO. Note: p38 experimental profiles are available only for WT, TRAF6 KO and TRADD KO. Experimental details and data are found in references [7,8,11,21]. ImageJ was used to estimate the intensities of the activation dynamics (Additional file 1: Table S1) for each molecule in each condition relative to wildtype peak activation values found in Additional file 1: Figure S2. IκBα phosphorylation refers to NF-κB activation throughout the text.

The parameters of the initial model (rate constants, or the elements of Jacobian matrix ***J***, Materials and Methods) were estimated by fitting the simulation profiles with experimental profiles of signaling molecules where data is available. We obtained published semi-quantitative experimental profiles of IκBα phosphorylation (Ν F-κΒ activation) and p38 (MAP kinase) activation in wildtype and various genetically mutant MEFs generally treated with 10 ng/mL of TNF (Figure 2A, Additional file 1: Figure S2 and Table S1) [7,8,11,21]. (Note that the kinetics of other MAP kinases, JNK and ERK, were also similar to p38 [7,8,11,21]. Thus, we used p38 as a representative MAP kinase for our investigation).

The parameter values were selected by using Genetic Algorithm [26] module in COPASI software [23] to fit the experimental profiles (Figure 2A, WT). Following, we performed sensitivity analysis (Materials and Methods) of the model parameters and found them to be robust to a small degree of uncertainty to their values (Additional file 1: Table S2). As a further validity of the parameter values, we tested the wildtype model in other conditions, namely TRAF2 KO, TRAF5 KO, TRAF2/5 double KO, TRAF6 KO, RIP1 KO and TRADD KO (Figure 2B). (Note that *in silico* KOs were generated from the wildtype model by setting the activation parameter value of the KO molecule to null).

Remarkably, we were able to obtain a single set of model parameters (Table 1, reactions 1–29 and see Additional file 2 for the TNFR1 model A in SBML format), which could be used to simulate the semi-quantitative profiles of IκBα phosphorylation and p38 kinase activation in multiple experimental conditions. In wildtype, TRAF2 KO, TRAF5 KO and TRAF6 KO, the IκBα phosphorylation and p38 kinase activation reach peak values around 15 min and gradually decay at 30 min. Notably, TRAF6 KO shows enhanced IκBα phosphorylation and p38 kinase activation due to *Signaling Flux Redistribution* (*Response Rule 5*, Additional file 1: Figure S1) [18]. In the remaining conditions, the activation levels of both molecules are very weak (RIP1 KO and TRAF2/5 DKO) or absent (TRADD KO).

**Table 1:** TNFR1 model A

	**Reaction**			**Formula and parameters (s**^**-1**^**)**						**Remarks**
**1**^**1**^	TNFR1	→	TRADD	*k*_*1*_	*	TNFR1	*k*_*1*_	=	5e-3	Activation of TRADD by TNFR1
**2**^**2**^	TRADD	→	cIAP1/2	*k*_*2*_	*	TRADD	*k*_*2*_	=	2e-2	Formation of Complex 1 containing TRADD, cIAP1/2, TRAF2, TRAF5, RIP1 and the TAB/TAK complex
**3**^**3**^	cIAP1/2	→	TRAF2	*k*_*3*_	*	cIAP1/2	*k*_*3*_	=	1e-2	
**4**^**4**^	cIAP1/2	→	TRAF5	*k*_*4*_	*	cIAP1/2	*k*_*4*_	=	8e-3	
**5**^**5**^	TRAF2	→	RIP1	*k*_*5*_	*	TRAF2	*k*_*5*_	=	1e-3	
**6**^**5**^	TRAF5	→	RIP1	*k*_*6*_	*	TRAF5	*k*_*6*_	=	1e-3	
**7**^**6**^	TRADD	→	TRAF6	*k*_*7*_	*	TRADD	*k*_*7*_	=	2e-2	Activation of TRAF6 by TRADD
**8**^**5**^	TRAF6	→	RIP1	*k*_*8*_	*	TRAF6	*k*_*8*_	=	1e-4	Activation of RIP1 and TAK1 complex by TRAF6
**9**^**7**^	TRAF6	→	TAK1 complex	*k*_*9*_	*	TRAF6	*k*_*9*_	=	1.3e-4	
**10**^**8**^	RIP1	→	LUBAC	*k*_*10*_	*	RIP1	*k*_*10*_	=	7e-3	Complex 1 ubiquitination by LUBAC and SHARPIN
**11**^**9**^	RIP1	→	SHARPIN	*k*_*11*_	*	RIP1	*k*_*11*_	=	7e-3	
**12**^**7**^	LUBAC	→	TAK1 complex	*k*_*12*_	*	LUBAC	*k*_*12*_	=	1e-1	
**13**	SHARPIN	→	IKK complex	*k*_*13*_	*	SHARPIN	*k*_*13*_	=	1e-2	Activation of IKK complex by Complex 1
**14**	TAK1 complex	→	IKK complex	*k*_*14*_	*	TAK1 complex	*k*_*14*_	=	1e-1	
**15**^**10**^	IKK complex	→	IκBα	*k*_*15*_	*	IKK complex	*k*_*15*_	=	1e-2	Phosphorylation of IκBα by IKK
**16**	IκBα	→	NF-κBc	*k*_*16*_	*	IκBα	*k*_*16*_	=	8e-3	Degradation of IκBα forms NF-κB
**17**	NF-κBc	→	NF-κBn	*k*_*17*_	*	NF-κBc	*k*_*17*_	=	1.7e-2	Translocation of NF-κB to nucleus
**18**	TAK1 complex	→	MKK1/2	*k*_*18*_	*	TAK1 complex	*k*_*18*_	=	1.5e-3	Activation of MAP kinases kinases by TAK1 complex
**19**^**11**^	TAK1 complex	→	MKK3/6	*k*_*19*_	*	TAK1 complex	*k*_*19*_	=	1e-2	
**20**	TAK1 complex	→	MKK4/7	*k*_*20*_	*	TAK1 complex	*k*_*20*_	=	1e-2	
**21**	MKK1/2	→	ERK	*k*_*21*_	*	MKK1/2	*k*_*21*_	=	5e-3	Activation of MAP kinases
**22**^**12**^	MKK3/6	→	p38	*k*_*22*_	*	MKK3/6	*k*_*22*_	=	5e-3	
**24**	MKK4/7	→	JNK	*k*_*23*_	*	MKK4/7	*k*_*23*_	=	5e-3	
**24**	p38	→	p38n	*k*_*24*_	*	p38	*k*_*24*_	=	5e-2	Translocation of MAP kinases into nucleus
**25**	JNK	→	JNKn	*k*_*25*_	*	JNK	*k*_*25*_	=	5e-2	
**26**	ERK	→	ERKn	*k*_*26*_	*	ERK	*k*_*26*_	=	5e-3	
**27**	p38n	→	AP1	*k*_*27*_	*	p38n	*k*_*27*_	=	1e-2	Activation of AP1 by MAP kinases
**28**	ERKn	→	AP1	*k*_*28*_	*	ERKn	*k*_*28*_	=	1e-2	
**29**	JNKn	→	AP1	*k*_*29*_	*	JNKn	*k*_*29*_	=	1e-2	
**30**	AP1	→	GI promoter	*k*_*30*_	*	AP1	*k*_*30*_	=	1e-1	Promoter binding of AP1 and NF-κB for group I genes
**31**	NF-κBn	→	GI promoter	*k*_*31*_	*	NF-κBn	*k*_*31*_	=	5e-3	
**32**	G1 promoter	→	GI pre-mRNA	*k*_*32*_	*	GI promoter	*k*_*32*_	=	1e-2	Group I genes transcription, splicing (1 step) and decay
**33**	GI pre-mRNA	→	GI mRNA	*k*_*33*_	*	GI pre-mRNA	*k*_*33*_	=	5e-2	
**34**	GI mRNA	→	GI mRNA decay	*k*_*34*_	*	GI mRNA	*k*_*34*_	=	2e-3	
**35**^**13**^	AP1	→	GII promoter	*k*_*35*_	*	AP1	*k*_*35*_	=	1.1e-2	Promoter binding of AP1 and NF-κB for group II genes
**36**	NF-κBn	→	GII promoter	*k*_*36*_	*	NF-κBn	*k*_*36*_	=	4e-3	
**37**	GII promoter	→	GII pre-mRNA/1	*k*_*37*_	*	GII promoter	*k*_*37*_	=	2e-3	Group II genes transcription, splicing (2 steps) and decay
**38**	GII pre-mRNA/1	→	GII pre-mRNA/2	*k*_*38*_	*	GII pre-mRNA/1	*k*_*38*_	=	5e-2	
**39**	GII pre-mRNA/2	→	GII mRNA	*k*_*39*_	*	GII pre-mRNA/2	*k*_*39*_	=	5e-2	
**40**^**13**^	GII mRNA	→	GII mRNA decay	*k*_*40*_	*	GII mRNA	*k*_*40*_	=	1.2e-4	
**41**	AP1	→	GIII promoter	*k*_*41*_	*	AP1	*k*_*41*_	=	5e-3	Promoter binding of AP1 and NF-κB for group III genes
**42**	NF-κBn	→	GIII promoter	*k*_*42*_	*	NF-κBn	*k*_*42*_	=	1e-4	
**43**	GIII promoter	→	GIII pre-mRNA/1	*k*_*43*_	*	GIII promoter	*k*_*43*_	=	1e-1	Group III genes transcription, splicing (3 steps) and decay
**44**	GIII pre-mRNA/1	→	GIII pre-mRNA/2	*k*_*44*_	*	GIII pre-mRNA/1	*k*_*44*_	=	4e-4	
**45**	GIII pre-mRNA/2	→	GIII pre-mRNA/3	*k*_*45*_	*	GIII pre-mRNA/2	*k*_*45*_	=	1e-3	
**46**	GIII pre-mRNA/3	→	GIII mRNA	*k*_*46*_	*	GIII pre-mRNA/3	*k*_*46*_	=	2e-4	
**47**	GIII mRNA	→	GIII mRNA decay	*k*_*47*_	*	GIII mRNA	*k*_*47*_	=	2e-5	
**48**	***GI mRNA***	***→***	***X1***	*k*_*48*_	*	GI mRNA	*k*_*48*_	=	1e-5	Feedback processes via group I genes or NF-κB
**49**	***NF-κBn***	***→***	***X1***	*k*_*49*_	*	NF-κBn	*k*_*49*_	=	5e-1	Steps of the secondary feedback processes (cytosolic or autocrine signaling):
**50**	***X1***	***→***	***X2***	*k*_*50*_	*	X1	*k*_*50*_	=	2e-3	
**51**	***X2***	***→***	***X3***	*k*_*51*_	*	X2	*k*_*51*_	=	2e-3	
**52**	***X3***	***→***	***X4***	*k*_*52*_	*	X3	*k*_*52*_	=	2e-3	
**53**	***X4***	***→***	***X5***	*k*_*53*_	*	X4	*k*_*53*_	=	2e-3	
**54**	***X5***	***→***	***X6***	*k*_*54*_	*	X5	*k*_*54*_	=	2e-3	
**55**	***X6***	***→***	***X7***	*k*_*55*_	*	X6	*k*_*55*_	=	2e-3	• expression (e.g. translation)
**56**	***X7***	***→***	***X8***	*k*_*56*_	*	X7	*k*_*56*_	=	2e-3	• transport (e.g. secretion)
**57**	***X8***	***→***	***X9***	*k*_*57*_	*	X8	*k*_*57*_	=	2e-3	• signaling (e.g. receptor binding, activation of transcription factors)
**58**	***X9***	***→***	***X10***	*k*_*58*_	*	X9	*k*_*58*_	=	2e-3	
**59**	***X10***	***→***	***X11***	*k*_*59*_	*	X10	*k*_*59*_	=	2e-3	
**60**	***X11***	***→***	***X12***	*k*_*60*_	*	X11	*k*_*60*_	=	2e-3	
**61**	***X12***	***→***	***X13***	*k*_*61*_	*	X12	*k*_*61*_	=	2e-3	
**62**	***X13***	***→***	***X14***	*k*_*62*_	*	X13	*k*_*62*_	=	2e-3	
**63**	***X14***	***→***	***IκBα***	*k*_*63*_	*	X14	*k*_*63*_	=	2e-3	IκBα feedback activation
**64**	***X14***	***→***	***Y***	*k*_*64*_	*	X14	*k*_*64*_	=	1e-5	Group III feedback activation via transcription factor Y
**65**	***Y***	***→***	***GIII promoter***	*k*_*65*_	*	Y	*k*_*65*_	=	2e-3	

**(1–12)**: *in-silico* knock-out conditions are performed by setting parameter values (*k*_*i*_) to 0 for targeted reactions in TRADD KO **(1)**, cIAP1/2 KO **(2)**, TRAF2 KO **(3)**, TRAF5 KO **(4)** TRAF2/5 DKO **(3,4)**, RIP KO **(5)**, TRAF6 KO **(6)**, TAK1 complex KO **(7)**, LUBAC KO **(8)**, SHARPIN KO **(9)**, IκBα KO **(10)**, MKK3/6 KO **(11)** and p38 KO **(12). (13)** Kinetics of Group II mRNA transcription and decay processes were refitted after adding feedback (without feedback: *k*_*35 *_= 7e-3, *k*_*40 *_= 1.2e-5). Bold italic fonts (reactions 48–65) indicate additional feedback activation pathways required for group III continuous activation. * indicates the multiplication sign.

It is noteworthy that although there have been previous models on TNF signaling [24,27,28], to our knowledge, this is the first time a single model of TNF signaling with fixed parameter values recapitulates the proinflammatory signaling dynamics in multiple experimental conditions.

To compare our linear response model (TNFR1 model A) simulations with other models that contain more detailed descriptions of IKK [28] and MAPK [29] signaling, using higher order terms and Michaelis-Menten type kinetics, we developed an alternative TNFR1 model B incorporating the relevant reaction details (Additional file 1: Table S3). Notably, the simulations of TNFR1 models A and B show very similar dynamics for a fixed amount of TNF perturbation (Additional file 1: Figure S3). Thus, we concur that our linear response model can be appropriately used for further investigations.

### Simulating distinct TNF-induced gene expression patterns

**Figure 3:**
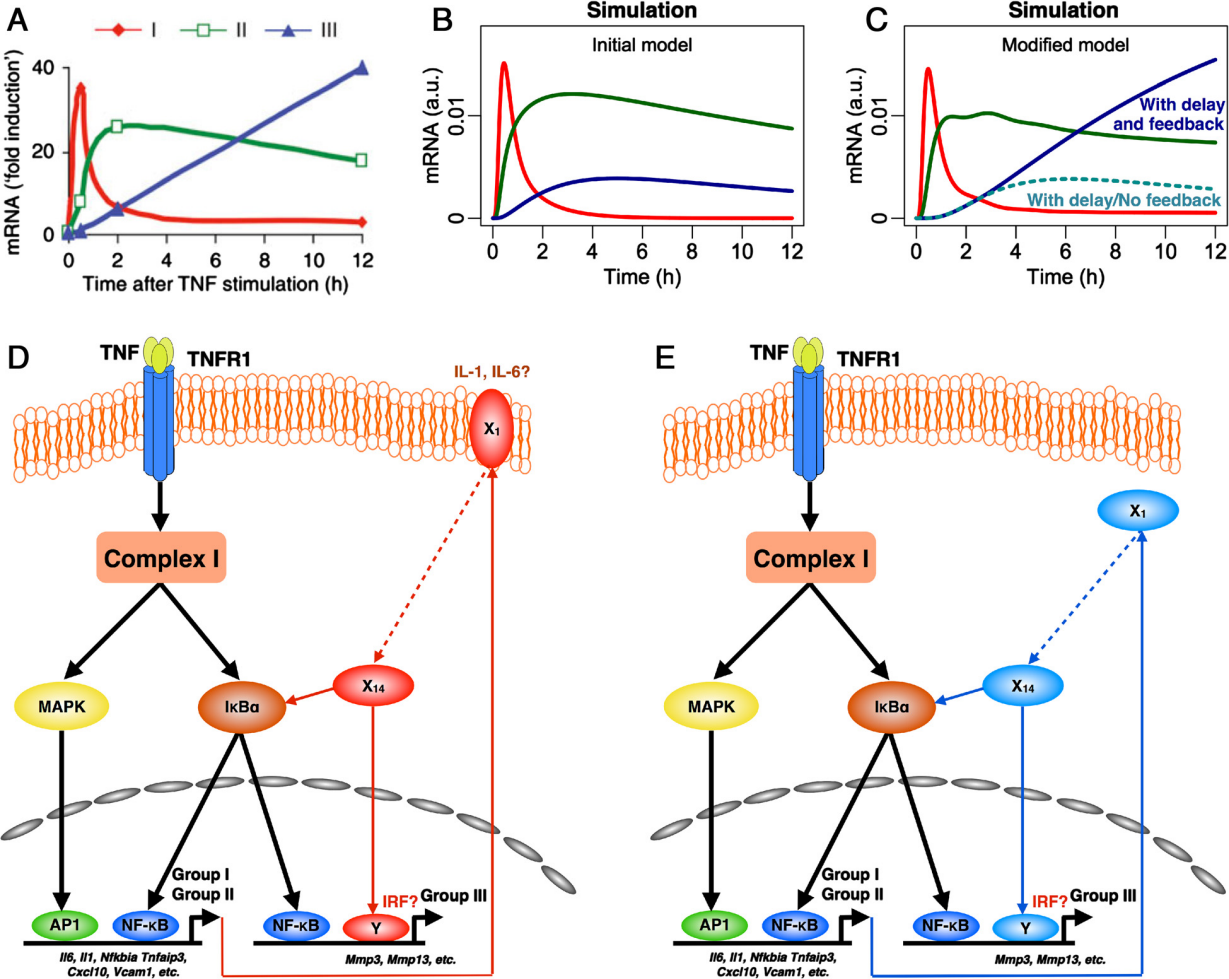
**Three distinct groups of TNF-activated genes. (A)** Average expression profiles of genes in groups I (red, peak at 0.5 h), II (green, 2h) and III (blue, 12h) in 3T3 fibroblasts stimulated with recombinant mouse TNF. Figure was reproduced from [12]. **(B)** Simulation profiles of the 3 groups of genes using the initial TNFR1 model. **(C)** Simulation of the modified TNFR1 model with transcriptional delay and novel feedback mechanisms (solid lines) or with transcriptional delay and without feedback mechanisms (dotted lines). **(D,E)** Proposed novel feedback pathway to provide additional signaling flux through translation of group I genes into proteins for autocrine signaling (red lines) **(D)** or cytosolic positive feedback (blue lines) **(E)**. Red and blue dotted lines indicate several intermediary molecular reactions (refer to Table 1). *X*_*1 *_to *X*_*14*_ refer to the novel intermediates included in the updated TNFR1 model (refer to Table 1, reactions 48–65), and *Y* refers to a novel transcription factor, such as interferon regulatory factor (IRF).

Next, we extended the TNFR1 model (we will now simply call TNFR1 model A as TNFR1 model) to simulate downstream proinflammatory gene expression dynamics. Recently, time-series high throughput microarray and quantitative real time PCR experiments on TNF simulated mouse 3T3 fibroblasts cells have revealed 3 distinct groups of upregulated gene expression patterns, with possibly corresponding distinct biological roles [12,30]. The groups were labeled into “early I”, “intermediate or middle II” and “late III” response, according to their time to reach peak expressions between 0.5-1, 2–3, and 6–12 h, respectively, after TNF stimulation (Figure 3A) [12,30,31]. Here, we extended the TNFR1 model to simulate the temporal profiles of the 3 groups of gene expressions.

According to our modeling approach, the time to peak activation can be controlled by reaction parameter values and/or the number of signaling intermediates [15,17-20]. Briefly, decreasing (increasing) the activation or transcription parameter value will show lower (sharper) gradients of formation part of the expression profiles. Alternatively, decreasing (increasing) the deactivation or decay parameter value will show lower (sharper) gradients of depletion part of expression profiles (*Response Rule 1*, Additional file 1: Figure S1). In addition, inserting intermediary reactions between transcription process and gene induction will increase delay for gene expression dynamics (*Response rule 2*, Additional file 1: Figure S1). The intermediates can represent the complexities of transcription process involving the pre-initiation, initiation, promoter clearance, elongation and termination [32], or post-transcriptional processes such as messenger RNA editing and splicing. Using this approach, the TNFR1 model was extended to simulate the temporal dynamics of groups I, II and III genes. Note that the *response rules* (Additional file 1: Figure S1) are used to modify an initial signaling topology only after all parameter space has been exhaustively searched, and a reasonable model fit is unable to be achieved [20].

Previous investigations on the 3 groups of genes have indicated distinct mechanisms for the differential dynamical response [12,30]. Hao and Baltimore have found lesser presence of AU Rich Element (ARE) region on the 3’UTR of group III genes, targeted by microRNAs and ARE-binding proteins (such as tristetraprolin) that enhance RNA decay processes. Hence, it was postulated as one possible reason for the lower decay response of group III genes compared with genes from groups I and II [12]. More recently, by studying the kinetics of pre-mRNA and mRNA, Hao and Baltimore observed delays in splicing of groups II and III genes compared to group I genes. The differential delays were suggested as another biological mechanism for the distinct gene profiles [30].

In our extended model, we, therefore, considered both mechanisms to reproduce the temporal profiles of the 3 groups of genes. Notably, our simulations of pre-mRNA and mRNA for all groups of genes matched the data of Hao and Baltimore for the first 60 min (Additional file 1: Figure S4). However, subsequently for 12 h, although the simulations of groups I and II genes were recapitulated, group III simulation was poor (Figure 3B, blue line). Specifically, reducing the parameter value for the decay term representing lower miRNA and ARE-binding proteins regulating decay processes (*Response rule 1*, Additional file 1: Figure S1), and adding intermediates (*Response rule 2*, Additional file 1: Figure S1) to provide delays in RNA splicing in our model were not sufficient to produce the continuous activation of group III genes (Figure 3C, cyan dotted line).

To overcome the shortfall in the model simulations, we hypothesized that novel activation or transcription term(s) (positive feedback) may be present to provide additional flux for the continuous increase in group III expressions (*Response rule 4*, Additional file 1: Figure S1). This could result from secondary post-transcriptional/translational mechanisms through i) autocrine signaling such as IL-1 [33], IL-6 [34] or TGF-β [35] signaling (Figure 3D), or ii) cytosolic feedback mechanisms specifically for group III genes [36] (Figure 3E). Thus, a novel feedback mechanism predominantly affecting the transcription of group III genes was added to the TNFR1 model (Table 1, equations 30–65 and Additional file 2).

The modified TNFR1 model with feedback mechanisms to group III genes produced simulations that matched all 3 groups of gene expression profiles (Figure 3A and C, solid lines). To scrutinize the feedback mechanism, we re-monitored the simulation profile of NF-κB for 6 hours (Additional file 1: Figure S5). The resultant profile mimics the damped oscillatory dynamics of NF-κB previously observed in murine fibroblasts [36]. Overall, these data suggest that low miRNA regulation and additional delay in RNA splicing are not sufficient to produce the continuous activation of group III genes, and that a novel transcription process, possibly through secondary post-transcriptional/translational autocrine signaling, such as IL-1 signaling or other novel feedback mechanisms that activate NF-κB, and not MAPK (Additional file 1: Figure S5), are required.

### Predicting key target for regulating proinflammatory response

Now that the TNFR1 model is able to successfully simulate the three groups of upregulated genes in wildtype, we investigated the significance and effect of removing or suppressing key intracellular signaling molecules for controlling proinflammatory response, *in silico*.

It is well known that TNFR1 signaling is enhanced in proinflammatory diseases and cancer [1-4]. To investigate which known molecules would be potential target to regulate the cell survival or proinflammatory activity, we performed *in silico* KOs of all possible signaling molecules within the TNFR1 model. In total, we simulated groups I, II and III dynamic gene expressions in 12 (TRADD, cIAP1/2, TRAF2, TRAF5, TRAF6, RIP1, SHARPIN, LUBAC, TAK1 complex (TAK1/TAB1/2), IκBα, MKK3/6 and p38) KO conditions and compared with wildtype profiles (Additional file 1: Figure S6).

**Figure 4:**
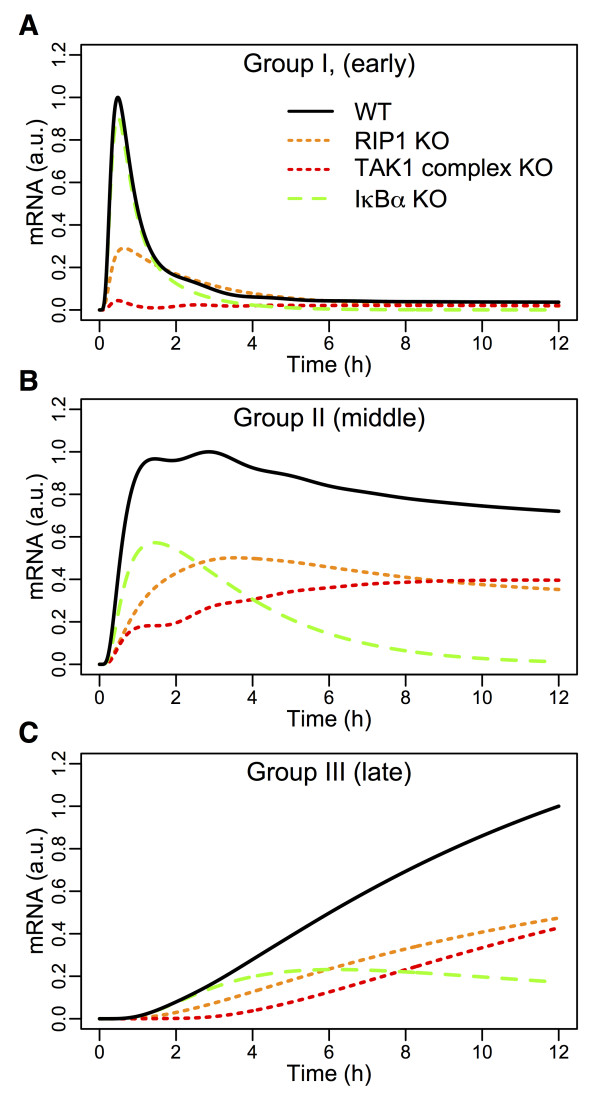
**The effects of *in silico *KOs on the expression profiles of the 3 groups of genes.** Simulated expression profiles of group I **(A)**, group II **(B)**, and group III **(C)** genes in 4 experimental conditions: wildtype (WT), IκBα KO, RIP1 KO and TAK1 complex KO for 12 hours using the modified TNFR1 model (with feedback). Predictions of RIP1 KO (orange curves) indicate suppression, but not abolishment, of all groups of gene expressions compared to wildtype (black curve).

Among the candidates, the removal of TAK1 complex or RIP1 produced the most noticeable downregulation of all 3 gene groups, which chiefly consist of well-known proinflammatory mediators (Figure 4). However, in TAK1 complex KO, our simulations show almost no induction for group 1 genes. The substantial impairment in gene expressions (> 90%) is usually detrimental to the general survivability of living cells, and this has been particularly demonstrated in TAK1-deficient mice [37,38]. RIP1, on the other hand, showed about 50-70% impairment compared to wildtype peak expressions. Our simulations, therefore, suggest that RIP1 is possibly a crucial single molecule target for controlling enhanced proinflammatory response due to TNFR1 signaling in proinflammatory disease conditions, such as in rheumatoid arthritis, without compromising the normal functioning of other cellular activities.

### Experimental inhibition of RIP1 downregulates proinflammatory genes in TNF stimulation

To verify the predictions of TNFR1 model simulations, we prepared corresponding MEF and BALB/3T3 cells treated with TNF in wildtype and in RIP1 suppression. Necrostatin-1 (Nec-1) was originally identified as a potent small molecule inhibitor of necroptosis or non-apoptotic cell death [39]. Further interests in Nec-1 led to its specificity towards the inhibition of RIP1 [40]. Although Nec-1 has recently been extensively studied, its effect on the expressions of groups I, II and III genes in TNF stimulation remains largely unknown. Therefore, here, we used Nec-1 to suppress RIP1 *in vivo*.

To check the effect of cell death by Nec-1, we compared MEF and BALB/3T3 cells treated with different doses of Nec-1 in the presence or absence of TNF (Additional file 1: Figure S7). The data revealed that Nec-1 has no substantial effect on cell death after 24 h incubation, and hence, could be tested for its efficacy on the 3 groups of genes. We next performed quantitative RT-PCR for a total of 10 genes: *Il6*, *Tnfaip3*, *Jun*, *Nfkbia* (group I), *Ccl7*, *Vcam1*, *Cxcl10* (group II), and *Mmp3*, *Mmp13*, *Enpp2* (group III). We intentionally included key proinflammatory mediators, genes of matrix metalloproteinase (*Mmp3*, *Mmp13*), which are known to degrade collagen in cartilage and thereby enhance rheumatoid arthritis and osteoarthritis progression [41-44].

**Figure 5:**
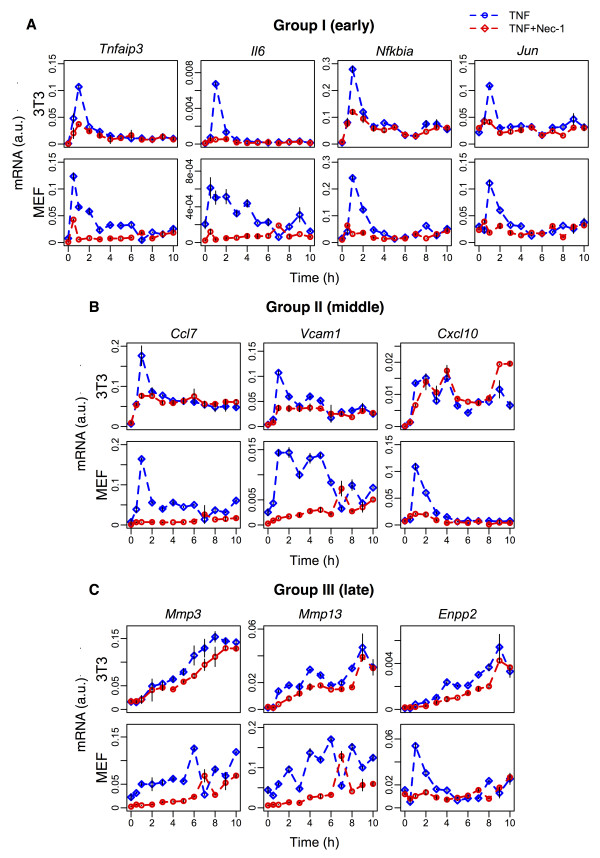
**Experimental verification of RIP1 inhibition through Nec-1.** Temporal gene expressions of groups I (*Tnfai3p, Il6*, *Jun*, *Nfkbia*) **(A)**, II (*Ccl7*, *Vcam1*, *Cxcl10*) **(B)**, and III (*Mmp3*, *Mmp13*, *Enpp2*) **(C)** genes in 10 ng/mL of TNF-stimulated BALB/3T3 (top panels) and MEF (bottom) cells, treated without (blue curves) and with (red curves) Nec-1. Nec-1 treatment was applied for 30 min before TNF stimulation. Curves indicate average profiles relative to *GAPDH* gene expression for *n* = *3* independent experiments, and error bars show mean values ± SD.

A previous study has shown that 30 μM of Nec-1 effectively inhibited RIP1 kinase activity [41]. Therefore, we investigated gene expressions for cells stimulated with 10 ng/mL TNF, in the presence or absence of 30 μM Nec-1 for a period of 10 hours with measurements made at least every hour (Figure 5). Remarkably, as predicted by the TNFR1 model, RIP1 inhibition by Nec-1 resulted in the suppression of all 3 groups of genes. The effect of suppressing RIP1 is significant for groups I and II genes in both MEF and BALB/3T3 cells, especially during the first 2–3 hours after stimulation. For group III genes, Nec-1 had more pronounced effect in MEF compared with BALB/3T3 cells. Overall, these results are consistent with the TNFR1 model predictions that suppressing RIP1 in TNF stimulation significantly impairs the activation of all 3 groups of genes.

## Discussion

TNF is a crucial cytokine that regulates myriad vital cellular processes. However, its levels are enhanced in major proinflammatory diseases. Here, to understand the TNF-induced proinflammatory signaling process, and to carefully regulate its dynamic response, a systems biology approach was adopted. We first developed a dynamic computational model using well-established publicly available experimental data of NF-κB, MAP kinase p38, and the average profiles of 3 groups of 180 upregulated genes in mouse fibroblast cells.

Despite the simplicity of using first-order response equations to simulate the profiles of the intracellular molecules, the computational model of TNFR1 recapitulated the experimental response in wildtype and several mutant conditions for NF-κB and p38 activations. This result is surprising, as we know that the innate immune response of TNF is highly complex. It is important to note here that there have been previous other computational efforts on NF-κB and MAPK signaling that had utilized detailed biochemical reactions modeling, to elucidate local properties of signal transduction, such as the ability of common molecules to produce distinct feedback mechanisms to different stimuli [24,45,46]. In our work, however, we have shown that even a simpler representation of the signal transduction pathways, through first order response equations and the law of mass conservation can reproduce experimental dynamics. This strongly indicates the presence of simple organizing rules governing the deterministic population average signaling response [47-52].

Next, through the analyses of downstream temporal gene expression profiles, the model suggests the presence of additional novel post-transcriptional/translational processes that is required for the continuous activation of group III genes. This result is additional to previous postulations, which had indicated that the continuous activation is due to lesser ARE region for group III genes leading to a very low decay process [12], and due to the presence of differential delays in the RNA splicing process [30]. Our model suggests that, on top of these effects, a novel time-delayed secondary transcriptional mechanism is required.

Literature survey indicates that the novel positive feedback processes could be a result of autocrine signaling, example through IL-1 or IL-6, or derive from a still unknown intracellular feedback mechanisms regulating mainly the promoter regions of group III genes. For example, the role of interferon regulatory factor (IRF) family in inducing *Ccl5* or RANTES expression, which belongs to one of the group 3 genes, is reported in a previous study [53], however, was not considered in the initial TNFR1 model. It is, therefore, necessary to perform further experimental work to confirm and elucidate the exact mechanisms for the continuous activations of group III genes.

On the other hand, for down-regulating TNF signaling, which is enhanced in several proinflammatory diseases and cancer, we performed the simulations for 12 *in silico* KOs of signaling molecules. The resultant simulations indicated that RIP1 is a major regulator of the 3 groups of upregulated gene expressions. To verify the result, we performed experiments on MEF and BALB/3T3 cells using Nec-1 as an inhibitor of RIP1. The measurement of 10 genes belonging to groups I (*Il6*, *Tnfaip3*, *Jun*, *Nfkbia*), II (*Ccl7*, *Vcam1*, *Cxcl10*) and III (*Mmp3*, *Mmp13*, *Enpp2*) all showed significant impairment with Nec-1 compared to wildtype.

Most importantly, the expressions of key proinflammatory genes such as *Il6, Vcam1, Ccl7, Mmp3*, *Mmp13*, enhanced in rheumatoid arthritis and osteoarthritis [41,44], were reduced. In particular are the levels of matrix metalloproteinase genes *Mmp3*, *Mmp13*, which are known to directly affect type II collagen in bone cartilages and degrade the extracellular matrix. Although recent therapeutics have been focusing on the specific regulations of MMPs [42-44,54], it remains to be seen what effect such treatments will have on other proinflammatory or vital genes.

In summary, our approach provides a systemic analysis of TNFR1 signaling, and suggests Nec-1 is potentially an important therapeutic target for effectively regulating major proinflammatory mediators in chronic diseases where TNF is overexpressed.

## Materials and methods

### Computational model

The model is based on perturbation-response approach [15,17-20]. The basic principle behind the approach is to induce a controlled perturbation of input reaction species of a system (TNFR1), and monitor the response of the activation/concentration levels of other output species (e.g. TAK1, p38, NF-κB, *Il6*, etc.) from steady-state. To briefly explain the principle, let a stable network consisting of *n* species be perturbed from the reference steady-state. In general, the resultant changes in the concentration of species are governed by the kinetic evolution equation [13,14]:

(1) $$\frac{\partial X_{i}}{\partial t}=F_{i}\left(X_{1},X_{2},..,X_{n}\right),\;i=1,..,n$$

where the corresponding vector form of equation 1 is $\frac{\partial X}{\partial t}=F\left(X\right)$. ***F*** is a vector of any non-linear function including diffusion and reaction of the species vector ***X =*** (*X*_*1,*_*X*_*2*_*, .., X*_*n*_), which represents activated concentration levels of reaction species. The response to perturbation can be written by ***X = X***_0_ + δ ***X***, where ***X***_0_ is the reference steady-state vector and δ ***X*** is the relative response from steady-states (δ ***X***_*t=0*_ = **0**).

The generally non-linear kinetic evolution equation 1 can be approximated or linearized by using Taylor series:

(2) $$\frac{\partial \delta X}{\partial t}=\frac{\partial F\left(X\right)}{\partial X}\;\delta X+\frac{\partial^{2}F\left(X\right)}{\partial X^{2}}\delta X^{2}+\ldots$$

As the general volume of perturbing substance is usually very small (order of 1%) compared to the total volume of cells that are perturbed [55], now consider a small perturbation around the steady-state in equation 2, in which higher-order terms become negligible and result in the approximation of the first-order term. In vector form ${\left(\frac{\partial \delta X}{dt}≅\frac{\partial F\left(X\right)}{\partial X}\right|}_{X=X_{0}}\;\delta X$ (note the change from partial derivative to total derivative of time), where the zeroth order term ***F*** (***X***_0_) = 0 at the steady-state ***X***_0_ and the *Jacobian* matrix, or linear stability matrix, is ${\left(J=\frac{\partial F\left(X\right)}{\partial X}\right|}_{X=X_{0}}$_._ The elements of ***J***, based on the initial activation topology, are chosen by fitting δ ***X*** with corresponding experimental profiles. Hence, the amount of response (flux propagated) along a biological pathway can be approximated using *first order mass-action response*, i.e. $\frac{d\delta X}{\mathrm{dt}}=J\delta X$. That is, the basic principle so far suggests that the response rate of species in a mass-conserved system at an initial steady-state can be approximated by first order mass-action response equations, given a small perturbation to one or more species.

Note that *Jacobian* matrix elements (or response coefficients) can include not only reaction information, but also spatial information such as diffusion and transport mechanisms. Thus, each species in the perturbation-response model can represent a molecule, a different modified state of a molecule (e.g. ubiquitinated state) or a molecular process such as diffusion, endocytosis, etc. That is, each species in the biological network does not necessarily represent a specific molecular species. For illustration, in a pathway *X*_*1*_ *→ X*_*2*_ *→ X*_*3*_ *→ X*_*4*_ *→ X*_*5*_, *X*_*1*_ to *X*_*5*_ can each be a different species or the same species at different stages in signaling, for example, *X*_*1*_ being internalized (becoming *X*_*2*_), transported to a different organelle (*X*_*3*_), ubiquitinated (*X*_*4*_) and become part of a protein complex (*X*_*5*_).

The complete SBML version of TNFR1 Models A & B are available in Additional file 2.

### Sensitivity analysis

We performed a sensitivity analysis to test the robustness of the optimized model parameters using the COPASI sensitivities module with default values. The variation in the response of signaling molecules/steps, *x*_*i*_(*t*), was analyzed when a small variation of each model parameter *k*_*j*_ was applied. The response sensitivity coefficient [56] of the *i*^th^ molecule with regard to the *j*^th^ parameter is defined by

(3) $$R_{i,j}=\frac{\partial x_{i}\left(t\right)}{\partial k_{j}}\frac{k_{j}}{x_{i}\left(t\right)}$$

The obtained values, *R*_*i,j*_ are then scaled, to reflect the relative changes in response, such as a change of *p*% in the value of parameter *k*_*j*_, results in a *R*_*i*_*,*_*j*_·*p*% change in the value of the peak activation of the *i*^*th*^ molecule. The response sensitivity coefficients of p38, IκBα, and groups I, II and III genes were obtained at peak time (*t* = 15 min for p38 and IκB, 30 min, 2 h and 12 h for groups I, II and III respectively, see Additional file 1: Table S2).

### Experiments

#### ***Reagents and cell culture***

Recombinant mouse TNF was purchased from R&D systems. Necrostatin-1 was purchased from Merck Millipore. 3T3 cells were obtained from JCRB cell bank. 3T3 and MEF were grown in DMEM (Nissui Seiyaku Co.) containing 10% calf serum, 100 U/mL of penicillin at 37°C in a 5% CO_2_ humidified atmosphere.

#### ***Evaluation of cell survival by 3-(4,5-dimethylthiazol-2-yl)- 2,5-diphenyltetrazolium bromide (MTT) assay***

The sensitivity of cells to hyperosmotic stress was measured with the MTT colorimetric assay in 96-well plates. Cells (2 × 10^4^) were inoculated in each well and incubated for 24 h. Thereafter, 50 μL of MTT (2 mg/mL in PBS) was added to each well and the plates were incubated for a further 2 h. The resultant formazan was dissolved with 100 μL of dimethyl sulfoxide after aspiration of culture medium. Plates were placed on a plate shaker for 1 min and then read immediately at 570 nm using TECAN microplate reader with Magellan software (Männedorf, Switzerland).

#### ***Real-time PCR analysis***

Total cellular RNA was extracted from cells using the TRIzol reagent according to the manufacturer’s instructions (Invitrogen). One microgram of RNA was reverse-transcribed using a first-strand cDNA synthesis kit (ReverTra Aceα; Toyobo). Quantitative real-time PCR was performed using SYBR premix Ex Taq (Takara) on the Applied Biosystems StepOnePlus^TM^ according to the technical brochure of the company. RT-PCR primers designed in this study are listed in Additional file 1: Table S4. Quantitative measurements were determined using the ΔΔCt method and expression of GAPDH was used as the internal control. Melt curve analyses of all real-time PCR products were performed and shown to produce the sole DNA duplex.

## Abbreviations

TNF: Tumor necrosis factor; MAP: Mitogen-activated protein; NF-κB: Nuclear factor-κB; MEF: Murine embryonic fibroblast; KO: Knock out; RIP1: Receptor-interacting protein 1; Nec-1: Necrostatin-1; TNFR: TNF receptor; DD: Death domain; TNFRSF: TNFR superfamily; TRADD: Tumor necrosis factor receptor 1 associated death domain protein; cIAP: Cellular inhibitor of apoptosis proteins; LUBAC: Linear ubiquitin chain assembly complex; HOIL-1: Heme-oxidized iron regulatory protein 2 ubiquitin ligase-1; HOIP: HOIL-1-interacting protein; SHARPIN: SH3 and multiple ankyrin repeat domains protein-associated RH domain interacting protein; TAK1: Transforming growth factor β(TGFβ)-activated kinase 1; AP: Activating protein; FADD: fas-associated death domain protein; TRAIL: TNF-related apoptosis-inducing ligand; DKO: Double knock out; mRNA: Messenger RNA; JNKm: c-Jun N-terminal kinases; ERK: Extracellular signal-regulated kinase; IκB: Inhibitors of NF-κB; ARE: AU rich element; miRNA: micro RNA; IL: Interleukin; RT-PCR: Reverse transcription polymerase chain reaction; IRF: Interferon regulatory factor; MMP: Matrix metalloproteinase.

## Competing interests

The authors declare that they have no competing interests.

## Authors’ contributions

KH and KS conceptualized and designed the study. KH and ST performed the wet experiments. VP and KS performed the computational simulations. MT provided cells, reagents and discussions. KH and KS wrote the paper. All authors read and approved the final manuscript.

## Supplementary Material

Additional file 1: Figure S1 Response rules**. Figure S2**. Experimental raw data used for model fitting. **Figure S3.** Experimental vs. simulated profiles of IκBα and p38 activations in wildtype and mutant conditions using TNFR1 model B. **Figure S4**. Simulation of pre-mRNA and mRNA expression profiles of groups I, II and III genes. **Figure S5**. Simulation of NF-κB activation profiles with and without feedback mechanisms. **Figure S6**. The effects of *in silico* KOs on the expression profiles of groups I, II and III genes. **Table S1**. Estimation of the relative intensities of IκBα and p38 activation dynamics. **Table S2**. Sensitivity analysis of TNFR1 model A. **Table S3**. TNFR1 model B details. **Table S4**. List of primer sequences for RT-PCR. Click here for file

Additional file 2 TNFR1 models A & B in SBML format. Click here for file
