# Supplementary material for: A systems biology approach to suppress TNF-induced proinflammatory gene expressions
Source: Cell Commun Signal. 2013 Nov 7;11:84. doi: 10.1186/1478-811X-11-84 (PMC3832246; doi:10.1186/1478-811X-11-84)
Supplement: Additional file 1: Figure S1 — Response rules. Figure S2. Experimental raw data used for model fitting. Figure S3. Experimental vs. simulated profiles of IκBα and p38 activations in wildtype and mutant conditions using TNFR1 model B. Figure S4. Simulation of pre-mRNA and mRNA expression profiles of groups I, II and III genes. Figure S5. Simulation of NF-κB activation profiles with and without feedback mechanisms. Figure S6. The effects of in silico KOs on the expression profiles of groups I, II and III genes. Table S1. Estimation of the relative intensities of IκBα and p38 activation dynamics. Table S2. Sensitivity analysis of TNFR1 model A. Table S3. TNFR1 model B details. Table S4. List of primer sequences for RT-PCR. [file 1478-811X-11-84-S1.docx]

**A Systems Biology Approach to Suppress TNF-induced Proinflammatory Gene Expressions**

Kentaro Hayashi, Vincent Piras, Sho Tabata, Masaru Tomita and Kumar Selvarajoo^*^

**Supplementary Materials**

**Figure S1. Response rules.**

**Response rules. *Rule 1, Controlling flux***: Controlling the upstream parameter (*k_1_*) of a hypothetical molecule *X_2_* mostly affects the slope of the formation part of the expression profile. Alternatively, controlling the downstream parameter (*k_2_*) mainly modifies the expression profile’s depletion part. ***Rule 2, Time delay***: by comparing the time to reach peak activation, any time delay in target signaling molecule’s activation represents ‘missing’ cellular features such as directed transport machinery, protein complex formation, and novel molecular interactions. ***Rule 3, Feedforward flux***: A) *Rapid kinetics*: when simulation of a downstream molecule is noticeably quicker than experimental dynamics, B) *Similar kinetics*: when removing a molecule along a pathway does not completely abolish its downstream intermediates, C) *Delayed kinetics*: when removing a molecule along a pathway show significant delay. In all these cases, the superposition principle suggests a novel feedforward pathway with different number of intermediates. ***Rule 4, Feedback flux***: when a response profile shows multiple peaks or continuous increase of activation not following pulse perturbation response, this indicates feedback pathways such as posttranslational effect or secondary (autocrine/paracrine) signaling. ***Rule 5, Signaling Flux Redistribution (SFR)***: At pathway junctions, removing a molecule enhances the entire alternative pathways. ***Rule 6, No SFR***: At pathway junctions, removing a molecule does not enhance the alternative pathway, suggesting novel i) intermediate(s) between the removed molecule and the pathway junction or ii) pathway link between the removed molecule and the alternative pathway. ***Rule 7, Differential flux***: quantifies each pathway branch by comparing activation levels between wildtype and mutants data. ***Rule 8, Reversible flux***: when a response profile show limiting decay that cannot be modeled by first-order decay, the presence of reversible step is expected to produce limiting decay. ***Rule 9, Non-linearity***: When complex dynamics is observed, the linear response approach breaks down, and non-linear approaches are needed.

**Figure S1. Response rules (continued).**

**Figure S1. Response rules (continued).**

**Figure S1. Response rules (continued).**

**Figure S1. Response rules (continued).**

**Figure S2. Experimental data used for model fitting.**

ImageJ was used to estimate the intensities of the activation dynamics for each molecule in each condition relative to wildtype peak activation values. We obtained the temporal activation profiles of signaling molecules after TNF stimulation (10 ng/mL) in (**A**) wildtype and TRADD KO from Ermolaeva *et al.* (*Nat Immunol* 2008, **9**:1037-1046, Fig. 1A) for p38 and IκBα, (**B**) wildtype and TRAF6 KO murine fibroblasts from Funakoshi-Tago *et al.* (*Cytokine* 2009, **45**:72-79, Fig. 2B and 3B) for p38 and IκBα, (**C**) wildtype and RIP1 KO from Devin *et al.* (*Immunity* 2000, **12**:419-429, Fig. 1A) for IκBα, and (**D**) wildtype, TRAF2 KO, TRAF5 KO and TRAF2/5 double KO from Tada *et al.* (*J Biol Chem* 2001, **276**:36530-36534, Fig. 1A) for IκBα. Figures adapted from their respective publications.


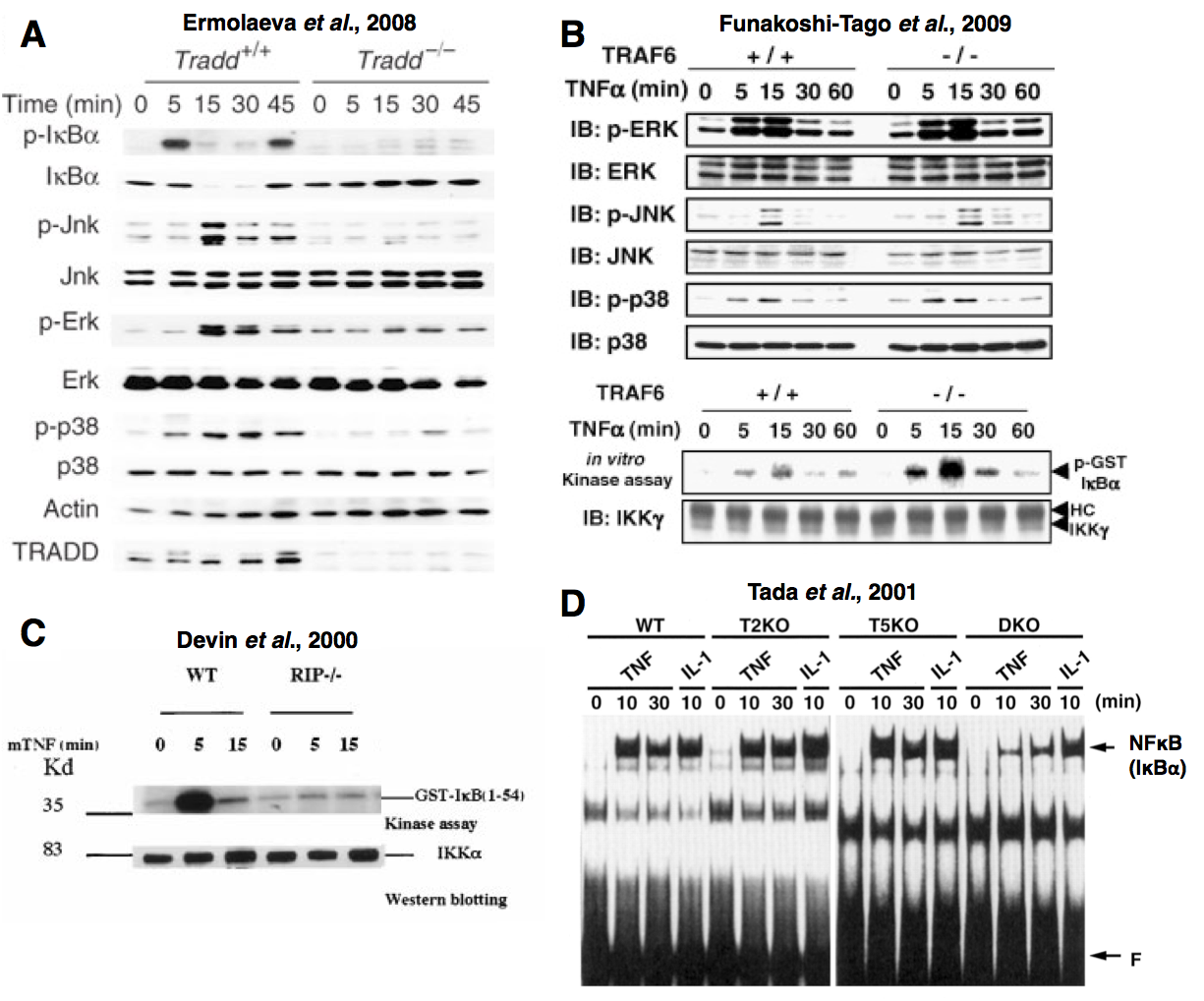


**Figure S3. Experimental vs. simulated profiles of IκBα and p38 activations in wildtype and mutant conditions using model B. (A)** Experimental profiles and **(B)** simulated profiles of TNFR1 model B (see Table S3) for IκBα (top panels) and p38 (bottom panels) activations.

**Figure S4. Simulation of pre-mRNA and mRNA expression profiles of the 3 groups of genes. Upper panels:** experimental pre-mRNA (red lines) and mRNA (blue lines) expression profiles in 3T3 cells of 3 representative genes from groups I, II and III, respectively, up to 60 minutes after TNF stimulation (10 ng/mL). **Lower panels:** simulations of pre-mRNA (red lines) and mRNA (blue lines) expressions using updated TNFR1 model. Upper panels are obtained from Hao S & Baltimore D (*Proc Natl Acad Sci U S A* 2013; **110**:11934-11939).

**Figure S5. Simulation of NF-κB activation profiles with and without feedback mechanisms.** (**A**) Simulations of nuclear NF-κB activation profiles up to 6 hours after TNF stimulation in wildtype condition without (dotted blue lines) feedback mechanisms and with feedback mechanism branched to IκBα (solid blue lines) or MAP kinases pathway (dotted orange lines) activation, are compared with experimental profiles obtained in TNF stimulated (10 ng/mL) 3T3 cells (red dots). (**B**) ImageJ was used to estimate the intensities of the activation dynamics relative to peak activation values from the data presented in Hoffmann *et al.* (*Science* 2002, **298**:1241-1245, Fig. 2E, adapted).

**Figure S6. The effects of *in silico* KOs on the expression profiles of the 3 groups of genes.** Simulated expression profiles of groups I **(A)**, II **(B)**, and III **(C)** genes in wildtype and 12 *in silico* KOs (see maintext) conditions for 12 hours using the modified TNFR1 model A (with feedback).

**Figure S7. Cell viability using Nec-1.** Cell sensitivity (MTT) assay for **(A)** 3T3 and **(B)** MEF cells treated in absence (light blue bars) or presence (brown bars) of 10 ng/mL of TNF, with indicated doses (0, 1, 5, 10, 15, 30 μM) of Nec-1 for 24 h. Average cell viability percentage for *n* = 3 independent experiments is shown. Error bars indicate mean values ± SD.

**Table S1. Estimation of the relative intensities of IκBα and p38 activation dynamics**.

| **IκBα** | **Time (min)** | **WT^1^** | **TRADD KO^1^** | **TRAF6 KO^2^** | **TRAF2 KO^3^** | **TRAF5 KO^3^** | **TRAF2/5 DKO^3^** | **RIP1 KO^4^** |
| --- | --- | --- | --- | --- | --- | --- | --- | --- |
|  | 0 | 0 | 0 | 0 | 0 | 0 | 0 | 0 |
|  | 5 | 0.3 | 0 | 0.6 |  |  |  | 0.1 |
|  | 10 |  |  |  | 0.9 | 1.05 | 0.15 |  |
|  | 15 | 1 | 0 | 1.9 |  |  |  | 0.1 |
|  | 30 | 0.45 | 0.1 | 0.75 | 0.4 | 0.5 | 0.22 |  |
|  |  |  |  |  |  |  |  |  |
| **p38** | **Time (min)** | **WT^1^** | **TRADD KO^1^** | **TRAF6 KO^2^** |  |  |  |  |
|  | 0 | 0 | 0 | 0 |  |  |  |  |
|  | 5 | 0.35 | 0 | 0.45 |  |  |  |  |
|  | 15 | 1 | 0 | 1.15 |  |  |  |  |
|  | 30 | 0.45 | 0 | 0.4 |  |  |  |  |

ImageJ was used to estimate the intensities of the activation dynamics for each molecule in each KO condition relative to wildtype peak activation values. Data was obtained from **(1)** Ermolaeva *et al.* (*Nat Immunol* 2008, **9**:1037-1046), **(2)** Funakoshi-Tago *et al.* (*Cytokine* 2009, **45**:72-79), **(3)** Tada *et al.* (*J Biol Chem* 2001, **276**:36530-36534), **(4)** Devin *et al.* (*Immunity* 2000, **12**:419-429).

**Table S2. Sensitivity analysis.**

The scaled response sensitivity coefficients, *R*, of each molecule/gene response at peak activation time (p38: 15 min, IκBα: 15 min, Group I: 30 min, II: 2 h, and III: 12 h) indicate the relative changes in response when individual parameters (rows) are varied, such as a change of *p*% in the value of parameter *k*, results in a *R*⋅*p*% change in the value of the peak activation of each molecule of interest. Absolute values of *R* higher than 1 indicate increasingly sensitive parameters.

**Table S3. TNFR1 model B**

|  | **Reaction** | | | **Formula and parameters** | | | | **Remarks** |
| --- | --- | --- | --- | --- | --- | --- | --- | --- |
| **1** | TNFR1 | → | TRADD | *k_1_* * TNFR1 | *k_1_* | = | 5e-3 | Activation of TRADD by TNFR1 |
| **2** | TRADD | → | cIAP1/2 | *k_2_* * TRADD | *k_2_* | = | 2e-2 | Formation of Complex 1 containing TRADD, cIAP1/2, TRAF2, TRAF5, RIP1 and the TAB/TAK complex |
| **3** | cIAP1/2 | → | TRAF2 | *k_3_* * cIAP1/2 | *k_3_* | = | 1e-2 |  |
| **4** | cIAP1/2 | → | TRAF5 | *k_4_* * cIAP1/2 | *k_4_* | = | 8e-3 |  |
| **5** | TRAF2 | → | RIP1 | *k_5_* * TRAF2 | *k_5_* | = | 1e-3 |  |
| **6** | TRAF5 | → | RIP1 | *k_6_* * TRAF5 | *k_6_* | = | 1e-3 |  |
| **7** | TRADD | → | TRAF6 | *k_7_* * TRADD | *k_7_* | = | 2e-2 | Activation of TRAF6 by TRADD |
| **8** | TRAF6 | → | RIP1 | *k_8_* * TRAF6 | *k_8_* | = | 1e-4 | Activation of RIP1 and TAB/TAK by TRAF6 |
| **9** | TRAF6 | → | TAK1 | *k_9_* * TRAF6 | *k_9_* | = | 1.3e-4 |  |
| **10** | RIP1 | → | LUBAC | *k_10_* * RIP1 | *k_10_* | = | 7e-3 | Complex 1 ubiquitination by LUBAC and SHARPIN |
| **11** | RIP1 | → | SHARPIN | *k_11_* * RIP1 | *k_11_* | = | 7e-3 |  |
| **12** | LUBAC | → | TAK1 | *k_12_* * LUBAC | *k_12_* | = | 1e-1 |  |
| **13** | SHARPIN | → | IKKγ | *k_13_* * SHARPIN | *k_13_* | = | 1e-2 | Activation of IKK complex by Complex 1 |
| **14** | TAK1 | → | IKKγ | *k_14_* * TAK1 | *k_14_* | = | 1e-1 |  |
| **15** | IKKγ +  IκBα/NF-κB | → | IκB complex | *k_15_* * IKKγ * IκBα/NF-κB | *k_15_* | = | 2.1e-3 | Formation of the IκB complex (IKKγ/IκBα/NF-κB) (and reverse step) |
| **16** | IκB complex | → | IKKγ +  IκBα/NF-κB | *k_16_* * IκB complex | *k_16_* | = | 8.9e-7 |  |
| **17** | IκB complex | → | IKKγ + IκBα + NF-κB | *k_17_* * IκB complex | *k_17_* | = | 2e0 | Dissociation of the IκB complex into IKKγ, phosphorylated IκBα and NF-κB |
| **18** | IκBα | → | IκBα degradation | *k_18_* * IκBα | *k_18_* | = | 1.7e-2 | Degradation of IκBα |
| **19** | IKKγ | → | IKKγ degradation | *k_19_* * IKKγ | *k_19_* | = | 4.6e-3 | Degradation of IKKγ |
| **20** | NF-κB | → | NF-κBn | *k_20_* * NF-κB | *k_20_* | = | 1.5e-2 | Translocation of NF-κB to nucleus |
| **21** | TAK1 + TAB | → | TAK1/TAB | *k_21_* * TAK1 * TAB | *k_21_* | = | 1e-2 | Formation and degradation terms for the TAK1/TAB complex |
| **22** | TAK1/TAB | → | TAK1/TAB degradation | *k_22_* * TAK1/TAB | *k_22_* | = | 7.1e-2 |  |
| **23** | MKK | → | MKKp | *k_23_* * (MKK * TAK1/TAB)  (*K_23_* + MKK) | *k_23_* | = | 2.7e-2 | Activation (double phosphorylation) of MAP kinase kinases (MKKs, e.g. MKK3/6) by the TAK1/TAB complex |
|  |  |  |  |  | *K_23_* | = | 5.9e-2 |  |
| **24** | MKKp | → | MKK | *V_24_* * (MKKp)  (*K_24_* + MKKp) | *V_24_* | = | 5.7e-4 |  |
|  |  |  |  |  | *K_24_* | = | 2.9e-2 |  |
| **25** | MKKp | → | MKKpp | *k_25_* * (MKKp * TAK1/TAB)  (*K_25_* + MKKp) | *k_25_* | = | 2.6e-2 |  |
|  |  |  |  |  | *K_25_* | = | 1.9e-7 |  |
| **26** | MKKpp | → | MKKp | *V_26_* * (MKKpp)  (*K_26_* + MKKpp) | *V_26_* | = | 3.8e-4 |  |
|  |  |  |  |  | *K_26_* | = | 9e-2 |  |
| **27** | MAPK | → | MAPKp | *k_27_* * (MAPK * MKKpp)  (*K_27_* + MAPK) | *k_27_* | = | 6.1e-2 | Activation (double phosphorylation) of MAP kinases (MAPKs, e.g. p38) by MKKs |
|  |  |  |  |  | *K_27_* | = | 3.9e-1 |  |
| **28** | MAPKp | → | MAPK | *V_28_* * (MAPKp)  (*K_28_* + MAPKp) | *V_28_* | = | 9.7e-5 |  |
|  |  |  |  |  | *K_28_* | = | 5.2e-4 |  |
| **29** | MAPKp | → | MAPKpp | *k_29_* * (MAPKp * MKKpp)  (*K_29_* + MAPKp) | *k_29_* | = | 2.5e-1 |  |
|  |  |  |  |  | *K_29_* | = | 9.9e-7 |  |
| **30** | MAPKpp | → | MAPKp | *V_30_* * (MAPKp)  (*K_30_* + MAPKp) | *V_30_* | = | 2.6e-6 |  |
|  |  |  |  |  | *K_30_* | = | 4.4e-1 |  |
| **31** | MAPKpp | → | MAPKn | *k_31_* * MAPKpp | *k_31_* | = | 8.2e-2 | Translocation of MAPKs into nucleus |
| **32** | MAPKn | → | AP1 | *k_32_* * MAPKn | *k_32_* | = | 1e-2 | Activation of AP1 by MAPKs |

Initial concentrations (nmol.mL^-1^): [TNFR1]*_t_*_=0_ = 1, [TAB]*_t_*_=0_ = 1, [MAPK]*_t_*_=0_ = 46, [MKK]*_t_*_=0_ = 52, [IκB/NF-κB]*_t_*_=0_ = 5. Units: *K_i_* in nmol.mL^-1^, *V_i_* in nmol.mL^-1^.s^-1^, *k_i_* in s^-1^ except *k_15_* and *k_21_* in mL.nmol^-1^.s^-1^ Colored rows indicate IKK (orange) and MAPK (light purple) modules adapted from Cho *et al.* (*Genome Res* 2003, **13**:2413-2422) and Kholodenko (*Eur J Biochem* 2000, **267**:1583-1588) respectively. Parameters and initial concentrations were determined through automated fitting of wildtype model using Genetic Algorithm optimization module in COPASI software.

**Table S4. List of primer sequences for RT-PCR**

| **Name** | **Species** | **Primer name** | **Sequence** |
| --- | --- | --- | --- |
| *Tnfaip3***^1^** | mouse | A20_F | GAACAGCGATCAGGCCAGG |
|  |  | A20_R | GGACAGTTGGGTGTCTCACATT |
| *Il6***^1^** | mouse | IL6_F | TAGTCCTTCCTACCCCAATTTCC |
|  |  | IL6_R | TTGGTCCTTAGCCACTCCTTC |
| *Nfkbia***^1^** | mouse | I*κ*Ba_F | CTGCAGGCCACCAACTACAA |
|  |  | I*κ*Ba_R | CAGCACCCAAAGTCACCAAGT |
| *Jun***^1^** | mouse | Jun_F | ACTCGGACCTTCTCACGTC |
|  |  | Jun_R | CGGTGTAGTGGTGATGTGCC |
| *Ccl7***^1^** | mouse | CCL7_F | GCTGCTTTCAGCATCCAAGTG |
|  |  | CCL7_R | CCAGGGACACCGACTACTG |
| *Vcam1***^1^** | mouse | Vcam1_F | AGTTGGGGATTCGGTTGTTCT |
|  |  | Vcam_R | CCCCTCATTCCTTACCACCC |
| *Cxcl10***^1^** | mouse | Cxcl10_F | AGGACGGTCCGCTGCAA |
|  |  | Cxcl10_R | CATTCTCACTGGCCCGTCAT |
| *Mmp3* | mouse | mmp3_F | CTCGTGGTACCCACCAAGTC |
|  |  | mmp3_R | AGTCCTGAGAGATTTGCGCC |
| *Mmp13* | mouse | mmp13_F | CTTCTGGCACACGCTTTTCC |
|  |  | mmp13_R | ATCCAGACCTAGGGAGTGGC |
| *Enpp2* | mouse | Enpp2_F | ACTCCGAGCAGCCTGATTTT |
|  |  | Enpp2_R | CCGGAGTAAGAGGTGAGCCA |

**(1)** Sequences obtained from Hao S & Baltimore D (*Nat Immunol* 2009; **10**:281-288).
